# Mass Transport in Osmotically Driven Membrane Processes

**DOI:** 10.3390/membranes11010029

**Published:** 2021-01-01

**Authors:** Peng Xie, Tzahi Y. Cath, David A. Ladner

**Affiliations:** 1 Department of Environmental Engineering and Earth Sciences, Clemson University, Clemson, SC 29625, USA; pxie@g.clemson.edu; 2 Department of Civil and Environmental Engineering, Colorado School Mines, Golden, CO 80401, USA; tcath@mines.edu

**Keywords:** forward osmosis, pressure retarded osmosis, computational fluid dynamics (CFD), feed spacer, 2D and 3D simulation, desalination, water treatment

## Abstract

Forward osmosis (FO) and pressure retarded osmosis (PRO) are the two operational modes for osmotically driven membrane processes (ODMPs). ODMPs have gained increasing popularity in the laboratory over the years; however, OMDPs have not been applied in very many cases at full scale because they are still emerging technologies that require further development. Computational fluid dynamics (CFD) modeling coupled with solute transport evaluation provides a tool to study hydrodynamics and concentration polarization in FO and PRO. In this study a series of models were developed to predict water flux. The simulation results of empty-channel (with no feed spacer) membrane cells were verified by comparison with experimental results, showing that CFD simulation with solute transport is a reliable tool. Ensuing 2D and 3D models were built to study the impact of feed spacers on the velocity and concentration distribution inside the flow channels, and investigate whether the presence of spacers would enable enhancement of water flux. The results showed that spacers could change the concentration and velocity profile and they could reduce or enhance water flux depending on the inlet flow velocity and distance between the membrane and spacer.

## 1. Introduction

Osmotically driven membrane processes (ODMPs) utilize an osmotic pressure difference to transfer water through a semipermeable membrane [1,2,3]. Forward osmosis (FO) and pressure retarded osmosis (PRO) are the two main OMDPs [4]. In FO, the active layer of the membrane faces the feed solution and the goal is usually to concentrate constituents in the feed or dilute the draw solution. In PRO, the active layer faces the draw solution [5] and the goal is usually to convert the pressure that builds up on the draw-solution side into useful energy. ODMPs have gained considerable attention due to the promises of wastewater purification or seawater desalination with FO [6,7,8] and energy production with PRO [9,10]. The main advantage of FO for water purification is that it operates at low hydraulic pressure, which is unlike other salt-rejecting membrane processes such as reverse osmosis (RO) and nanofiltration. FO may also be used in a treatment train with other unit processes like RO [1].

The study of ODMPs has distinct differences compared to more conventional and well-understood pressure driven membrane processes such as RO. In RO, concentration polarization (CP) almost exclusively exists in the feed channel near the active layer of the membrane, known as external CP (ECP) [11,12]; however, in FO and PRO two types of CP exist: ECP on the active layer and support layer of the membrane, and internal CP (ICP) within the membrane support layer [13,14]. ICP is more difficult to control [15,16] and is the primary hindrance to high water flux in FO [5,17]. Many studies have confirmed that ICP can be reduced by making the membrane support layer thinner and more permeable [4,12,18,19,20]; however, further material development is needed.

ECP can be reduced by changing the fluid characteristics (e.g., viscosity and temperature), increasing flow velocity, or enhancing mixing (e.g., with spacers that increase turbulence). Studies on reducing ECP in ODMPs with spacers are very few compared to the plethora of studies available for spacers in RO systems [9,21,22]. It is anticipated that ECP will be similar in ODMPs and RO because it is driven by flux values that may be similar, but it is important to model both ECP and ICP concurrently in ODMPs because the driving force for flux depends on salt concentrations outside and inside the membrane support. Understanding the impacts of spacers on ODMPs could advance the development of spiral wound modules or other configurations for ODMPs, making them more economical and feasible, similar to the historical development of RO.

Some experimental efforts have been made to study the impact of feed spacers on FO. Zou et al. [23] found that using a spacer on the feed side of the FO membrane both reduced the ECP and lowered the fouling propensity. Others have observed the opposite effect, where the spacer increased the fouling propensity [24]. Linares et al. [22] found that using thicker feed spacers could reduce flux decline caused by biofouling in FO. Zhang et al. [16] reported that placing the feed spacer in the draw solution channel in contact with the membrane while the feed spacer in the feed channel is placed far away from the membrane could enhance flux for FO. However, their spacer was much thinner (0.8 mm) than the channel height (3.5 mm), which would not be possible in practice (such as in a spiral wound module) where the spacer holds the membrane leaves apart and thus the channel height is about the same as the spacer thickness. These experimental results are useful, but it is difficult to experimentally observe and quantify the hydraulic and mass transfer conditions inside the system at the high spatial resolution required to understand ECP. Computational methods are one way to make progress in this field.

Computational fluid dynamics (CFD) could be useful in studying FO and PRO because it can calculate and visualize the fluid flow inside the membrane system, which is difficult in experimental work [19,20,25,26,27,28,29,30]. Extending CFD models by including solute transport enables us to also study mass transfer. Modeling is helpful for designing spacers in next-generation FO and PRO modules; it is more economical to evaluate new designs in silico first as a screening tool, then to take only the most promising designs into fabrication and lab testing. Sagiv et al. [13] developed a 2D finite element method (FEM) model to study FO with several different types and concentrations of draw solutions. McCutcheon et al. [15] developed a 2D analytical model to study the coupled effects of ECP and ICP on water flux. Gruber et al. [31] developed a 3D CFD model to study the FO process and optimize module design.

All of the above-cited studies simulated modules without spacers; thus, CFD studies of ODMPs with spacers are very limited. The only literature found at the time this work was performed was by Park et al. [21]—they studied the impact of spacer configuration on ECP in FO and PRO in a 2D simulation by combining both ECP and ICP into a single parameter. They reported that the spacer could mitigate ECP in both PRO and FO; however, the benefit of using spacers may be diminished because local water flux is blocked where the spacer touches the membrane [21]. Such a 2D model is useful, but 2D simulations are not able to fully model the effects of flow angle and spacer orientation [6,16,24,32]; for this, 3D simulations are needed. The main drawback to 3D simulations of spacer-filled FO and PRO modeling is that they are more difficult to converge and require much more computational power and time. One 3D CFD simulation of FO was reported by Gruber et al. [31] where flux results from simulation were verified using experimental results, but their focus was not on spacers. An important conclusion was that analytical modeling approaches severely underestimated the ECP and the numerical model was more accurate. In another study spacers were evaluated, but the flux was set as a parameter and not driven by the concentration gradient across the membrane [30]. A more recent review discusses further progress in the field [33].

In this study, a series of models were developed to predict water flux in ODMPs. The simulation results of empty-channel (with no feed spacer) membrane cells were verified by comparison with experimental results from the literature. Then both 2D and 3D spacer-filled models were built to investigate the impact of feed spacers on hydrodynamics and mass transfer inside the membrane channel. The study also evaluated the degree to which feed spacers can potentially enhance water flux.

A key to adequately simulating ODMPs is to fully couple the hydrodynamics with solute mass transport. This has been done previously for simulations of RO systems [32], but in RO the coupling only needs to be in one domain, the feed channel above the membrane surface. The problem becomes weightier when two additional domains (the membrane support layer and the draw-solution channel) need to be modeled simultaneously with the feed channel. However, in addition to that weight, the problem becomes much more complex because the three domains are coupled with each other. For example, water and solute entering the membrane support layer needs to be consistent with the water and solute leaving the feed channel. Accomplishing this full coupling of hydrodynamics and solute transport across the three domains was one of the main contributions of this work.

## 2. Materials and Methods

### 2.1. Membrane Characteristics

Two types of membranes were simulated in this study: a polyamide thin-film composite (TFC) membrane and a cellulose asymmetric membrane. These were used in a round-robin study of ODMP testing methods [4]. The TFC membrane was from Oasys Water (Boston, MA) and the asymmetric membrane was a from Hydration Technology Innovation (HTI, Albany, OR). Most membrane characteristics such as water permeability and salt permeability were as reported in the round-robin study [4]. For the TFC membrane, the thickness of the support layer (40 µm) was estimated from information in a patent [34]. The hydraulic conductivity (*κ* = 2.34 x 10^–15^ m/s), porosity (*ε* = 0.41), and tortuosity (*τ* = 1.7) of the support layer were obtained from Tiraferri et al. [35] and Sagiv et al. [13]. For the asymmetric membrane, the thickness of the support layer (100 µm) and the porosity/tortuosity relationship (*ε/τ* = 0.163) were obtained from Phillip et al. [36].

### 2.2. Equations for Fluid Flow and Mass Transfer

Water flow in the feed and draw solution channels was calculated by Equations (1) and (2):

(1) ∇·u=0

(2) $$\rho \left(u\cdot \nabla u\right)=-\nabla P+\mu \nabla \cdot \left(\nabla u+{\left(\nabla u\right)}^{T}\right)]$$

where *u* is the velocity, *ρ* is the density of the solvent, *P* is the pressure, and *μ* is the dynamic viscosity of the solvent.

The fluid flow within the membrane support layer was treated as fluid flow in a porous medium calculated with the Darcy–Forchheimer law, which is solved numerically according to Equation (3):

(3) $$(\frac{\rho }{\varepsilon }\left(u\cdot \nabla \right)\frac{u}{\varepsilon })=\nabla \cdot \left[-P+\frac{\mu }{\varepsilon }\left(\nabla u+{\left(\nabla u\right)}^{T}\right)-\frac{2\mu }{3\varepsilon }\left(\nabla \cdot u\right)\right]-\left(\mu k^{-1}+\beta_{F}\left|u\right|\right)u$$

where *u* is velocity in the membrane support layer, *ε* is the support layer porosity, *k* is the support layer water permeability, and *β_F_* is the Forchheimer coefficient:

(4) $$\beta_{F}=\frac{1.75}{\sqrt{150\varepsilon^{3}}}\;\cdot \frac{\varepsilon \rho }{\sqrt{k}}$$

With the addition of the Forchfeimer coefficient, Equation (3) takes into account the coupled effects at the interface of the free water channel and porous medium [37].

The flux of water through the membrane selective layer was calculated by:

(5) $$J_{w}=A_{F}\cdot \left(\pi_{d}-\pi_{f}\right)$$

where *J_w_* is the flux of water through the membrane, *A_F_* is the water permeability of the membrane at 293 K (room temperature), *π_d_* is the osmotic pressure on the draw solution side near the active layer of the membrane, and *π_f_* is the osmotic pressure at the feed solution side near the active layer of the membrane. Equation (5) is the same transport model used for pressure-driven salt-rejecting membrane processes, but with the applied pressure set to zero. Note that in full-scale implementation PRO would use applied pressure, but in the round-robin study [4] upon which our modeling work was based (see Section 2.3), no external pressure was applied; rather the PRO experiments were simply operated in the reverse direction of the FO experiments. In the model the active layer had no thickness, but was instead treated as a boundary between the feed channel and support layer computational domains.

The solute (salt) transfer in the membrane channel was calculated by:

(6) $$u\nabla c=D\nabla^{2}c$$

where *c* is the salt concentration and *D* is the diffusion coefficient in the membrane channel.

The solute transport in the membrane support layer was calculated by:

(7) $$u\nabla c=D_{e}\nabla^{2}c$$

where *D_e_* is the effective diffusion coefficient in the membrane support layer calculated by:

(8) $$D_{e}=\frac{\varepsilon }{\tau }D$$

The flux of salt through the membrane was obtained by

(9) $$J_{s}=B\cdot \Delta c$$

where *J_s_* (mol/s/m^2^) is the flux of salt through the membrane, *B* (m/s) is the salt permeability of the membrane, and *Δc* is the salt concentration difference across the selective layer, with the two concentration values taken immediately adjacent to the selective layer on each side.

Equations (1), (2), and (5) were implemented in COMSOL through the “Laminar Flow” module, while Equation (6) was integrated in the “Transport of Diluted Species” module. These are well-established modules for evaluating fluid flow and mass transport. The “Flow in Porous Media” and “Transport of Diluted Species in Porous Media” encompassed Equations (3), (4), (7)–(9), to study flow and mass transport in the membrane support layer, which is conceptualized here as a medium with homogeneous porosity.

**Figure 1:**
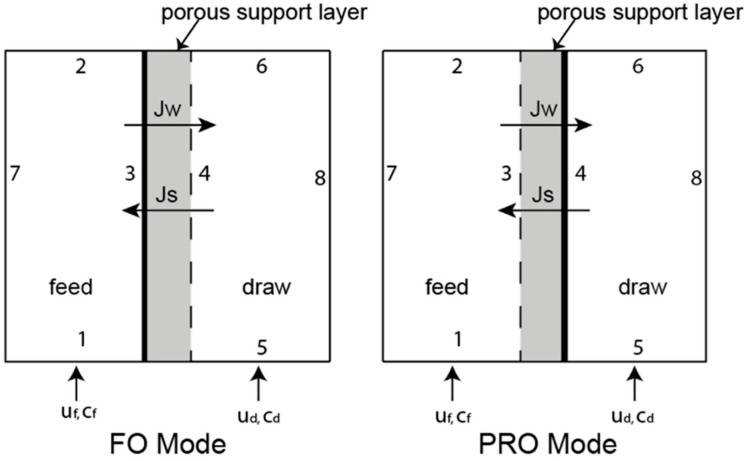
Flow-channel domains for two configurations: (**left**) FO mode in which the active layer (indicated by the bold vertical line) faces the feed-side; (**right**) PRO mode in which the active layer faces the draw solution channel. The porous support layer is denoted by the grey area. Inflow of water and salt concentration in the feed and draw solution are denoted *u_f_*, *u_d_*, *c_f_*, and *c_d_*, respectively. Fluxes of water and salt are denoted *J_w_* and *J_s_*, respectively. The boundaries are numbered 1 through 8.

The locations of the boundaries in the simulation of FO and PRO membrane channels are described in Figure 1 and Table 1. In the table, the locations where mass (water and salt) flow into and out of the simulation are indicated by “inlet” and “outlet,” respectively. Where a variable is given (e.g., “*u_f_*” for feed inlet velocity), the value of the variable is defined in the description of each scenario. Some of these values are fixed (*u_f_*, *u_d_*, *c_f_*, and *c_d_*), while others are calculated during the simulation (*J_w_* and *J_s_*). The pressure was set to zero (atmospheric) at the outlet of the feed and draw solution channels in all simulations. None of the simulations had significant longitudinal pressure drop because only short sections of membrane were modelled. This method allowed us to keep the hydraulic pressure constant and the flux variations seen in different simulations were caused only by differences in CP that changed the osmotic driving force. The method thus allowed us to study only ODMPs with no pressure-driven effects. Steady state models were used throughout this ODMP simulation work. Other details, including a depiction of the CFD mesh scheme and sensitivity analysis, can be found elsewhere [38].

### 2.3. Model Verification with Empty Flow Channels

The first step of this study was to build 2D CFD models, which had the same dimensions and operating conditions (flow channel dimensions, inlet velocity, concentration, and membrane type) as a set of experimental data. The data chosen were the result of a round-robin exercise where two membrane types were tested in empty channels by multiple laboratories to help establish standard protocols for performance evaluation [4]. The data set was considered a robust, validated set, useful for comparison with our simulation results. The CFD models were designed to calculate the water flux based on the calculated hydrodynamics and membrane characteristics in the membrane cell. Flux through the membrane was calculated based on the osmotic driving force created by the difference in salt concentration at the membrane walls determined by the simulation. This is noteworthy because many CFD studies of membrane processes use a fixed flux determined from experiments. Here the flux was predicted, rather than fitted to the experimental data.

The dimensions of the feed and draw solution channel were 77 mm long, 26 mm wide, and 3 mm deep. Two combinations of feed and draw solution concentrations were tested in the experiments [4]: (1) deionized water feed and draw solution concentration 1 M NaCl and (2) feed concentration 0.5 M NaCl and draw solution concentration 1.5 M NaCl. Both combinations were tested in FO and PRO mode. The crossflow velocity (*u_f_*) was 0.25 m/s.

### 2.4. Spacer Filled 2D and 3D Flow Channels

The model verification work discussed above was performed for simulations and experiments with empty flow channels. The modeling strategy was then used to evaluate spacer-filled flow channels. 2D simulations were primarily used; the benefit of 2D simulations is that the algorithms can converge on a solution more easily than in 3D. This allows for testing of a larger range of velocities than can be tested in 3D simulations. However, because 2D simulations are not able to show the flow and concentration in angles that are not along the flow direction, 3D models are beneficial. The results from a few 3D models were analyzed and compared with the 2D case to help increase our understanding of effects that may be altered when moving from 2D to 3D.

The geometry of a conventional mesh spacer is often described as a “fish net” formed from two layers of filaments with circular cross section [27,39]. In this depiction, the top layers of filaments are always touching the membrane surface; however, in reality, the cross sectional shape and thickness of the filaments is irregular [39]. In many studies, the cross-section is considered as elliptical [40,41,42]. Moreover, the filaments are not constantly in contact with the membrane; instead there is space between the membrane and the spacer in many locations.

In this study, we modeled a mesh spacer that resembled those used in practice, but with a geometry that could be easily modeled in both 2D and 3D. The mesh spacer was formed by two layers of filaments with elliptical cross sections. The ellipses were 0.5 mm high and 0.8 mm wide. The center-to-center distance of the filaments on the same layer was either 6 or 12 mm. Various membrane–filament distances were evaluated: 0, 0.01, 0.05, 0.1, 0.15, and 0.25 mm. For 3D simulations, filaments that touched the membrane caused sharp corners with acute, dead-end angles in the mesh that made it difficult for the models to converge on a solution, so the smallest 3D membrane–filament distance was set at 0.01 mm. The membrane channel height was 1 mm, so the 0.01 mm distance constitutes 1% of the membrane channel height. This results in a model where the spacer is close enough to almost eliminate flow between the spacer and the membrane, so it is in effect touching the membrane while still allowing the simulation to converge. The membrane–filament distance was constant along the length of the filament. Note that a 3 mm channel height was used previously to make comparisons with the experimental data, which were collected with membrane cells that had that channel height. For the spacer-filled models, a 1 mm channel height was chosen because it is closer to the typical depth of membrane channels in spiral wound modules.

**Figure 2:**
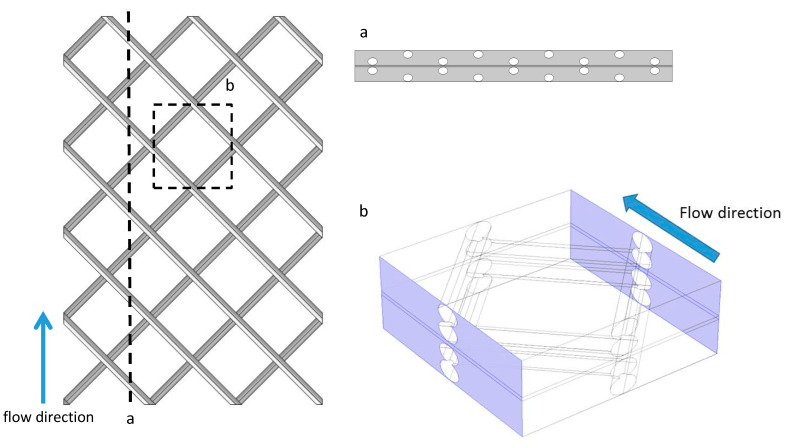
3D illustration of the mesh spacer (**left**). The dashed line indicates the location of the 2D model (**a**, top right). The dashed lined square indicates the location of the 3D model (**b**, bottom right). The location of periodic boundaries is marked in purple.

The geometry of the 2D models was taken as a cross sectional view of the 3D mesh spacer (Figure 2). The dimensions for feed and draw solution channels were 27 mm long by 1 mm high. The dimensions for the membrane support layer were 27 mm long and 0.1 mm high. The geometry of the 3D model was taken as a portion of the mesh spacer (Figure 2). Because running the 3D model required much more computational power, the dimension of the feed and draw solution membrane channels were limited to 6 mm long, 6 mm wide, and 1 mm deep. The dimensions of the membrane support layer were 6 mm long, 6 mm wide, and 0.1 mm deep. The boundary conditions were the same for both the 2D empty flow channel and the 2D spacer-filled flow channel.

Because part of the entrance of the 3D model is blocked by the feed spacer (Figure 2), the inlet boundary condition for the 3D model is flow rate (6 × 10^–7^ m^3^/s) instead of inlet velocity as in the 2D model. This ensures that the average velocities in both the 2D and 3D models are comparable. To reduce the impact of the boundary effects to the model, periodic boundary conditions are used in the boundaries of the channel that were parallel to the flow direction (Figure 2). It should be noted that the Reynolds number for empty flow channels in the laboratory setup was around 1500. Because the height of the channel was reduced from 3 mm to 1 mm and spacers were included, both the 2D and 3D models were unable to converge at the original 0.25 m/s flow velocity. After a series of trials, the maximum cross flow velocity for a fully coupled 3D FO model to converge was 0.01 m/s. To study the flow pattern inside the 3D FO flow channels under higher cross flow velocity, another series of 3D models was built, which set the water and salt fluxes to zero to make the model converge. Based on our results, the permeate flow rate was only up to 0.2% of the inlet flow rate, so neglecting the water flux should not make a significant impact on the flow patterns. It should be stressed that these zero-flux 3D models were only used to evaluate and visualize fluid flow patterns for the 0.15 m/s crossflow velocity case. Conclusions regarding the effects of spacers on ECP and water flux were derived from the 2D and low crossflow velocity 3D simulations.

## 3. Results and Discussion 

### 3.1. Modeling Verification

**Figure 3:**
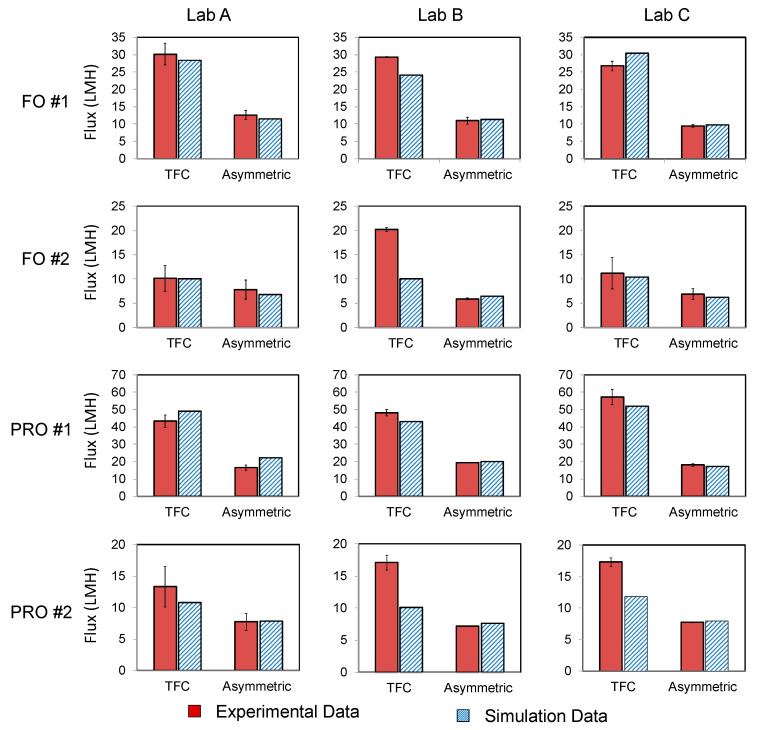
Comparison of water flux (L/m^2^/hr) from simulations and literature [4] conducted in FO and PRO modes (no hydraulic pressure). Labs A, B, and C were compared in the round-robin study [4]. In scenario #1, the feed was deionized water and the draw solution concentration was 1 M NaCl, while in #2 the feed concentration was 0.5 M NaCl and the draw solution concentration was 1.5 M NaCl.

The water flux calculated from the 2D simulations was compared with the data from the experimental study [4] (Figure 3). The error (*E*) between the simulation results and literature report was calculated using Equation (10):

(10) $$E=\;\frac{\left|J_{model}-J_{lab}\right|}{J_{lab}}\;\times 100\%$$

where *J_model_* is the flux obtained from the simulations and *J_lab_* is the flux obtained from lab experiments.

Results indicate that except for the FO #2 and PRO #2 scenarios for the TFC membrane, the average error was not more than 12%. In general, the error was smaller for the asymmetric membrane and the two smallest errors were observed in FO #1 (5%) and PRO #2 (3%) scenarios for the asymmetric membrane. The two largest average errors were observed in FO #2 (19%) and PRO #2 (30%) for the TFC membrane. It should be noted that those two scenarios also exhibited a large difference in experimental water flux among the labs—in FO #2 the water flux reported by laboratory B was much higher than the other two laboratories and the difference between experiments and simulations in that scenario was as high as 50%; in PRO #2 the water flux from laboratory A was lower than the other two laboratories for the TFC membrane. The standard deviation of each experimental result is summarized in Table 2. In general, the standard deviation of the experimental data for the asymmetric membrane was smaller than the standard deviation of the TFC membrane.

### 3.2. D Simulations with Feed Spacer-Filled Flow Channels

The results of the model verification exercise lend confidence to using this modeling framework for predicting performance in alternative scenarios. In this work we were particularly interested in evaluating the effects of spacers placed in the feed and draw solution channels. Because the simulations used the experimentally derived membrane characteristics (e.g., *A_f_* and *B*) and also used a full coupling of the hydrodynamics and mass transfer inside the membrane channel, the flux was predicted in the empty flow channel cases, rather than fitted. It then stands to reason that if the models can accurately predict the hydrodynamics and solute mass transport in spacer-filled flow channels, the flux prediction for those channels may also be reliable.

Parameters such as *A_F_* and *R* for simulations of FO with spacers were based on FO with asymmetric membranes in scenario #1 (deionized water feed and 1 M NaCl draw solution) reported by laboratory B in the round-robin study [4]. These were chosen because (as shown in Figure 3 and Table 2) they were the best match based on the difference between modeling and experiments (2%) and they had the lowest standard deviations (0.35 L/m^2^/hr). We simulated several spacers in both FO and PRO modes, but found that conclusions about hydrodynamic and mass transport effects were similar, so to make the discussion more concise we present only FO-mode simulation data here.

**Figure 4:**
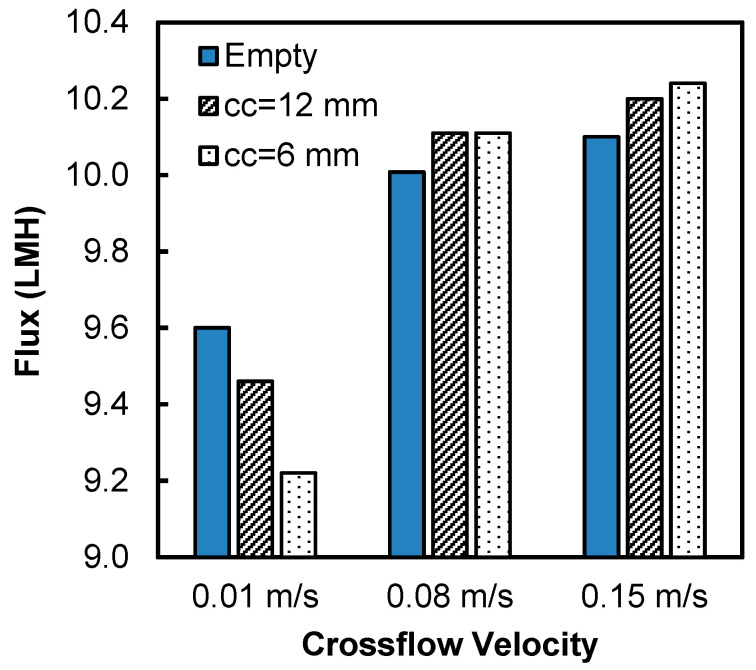
Water flux for empty and spacer-filled channels with spacers having center-to-center (cc) filament distances of 6 mm and 12 mm.

Two types of spacers with center-to-center distance of 6 mm and 12 mm were tested in this study. The water flux at three different inlet velocities (0.01, 0.08 and 0.15 m/s) for the spacer-filled flow channels were compared to the flux during operation with empty flow channels (no spacers) (Figure 4). The results show that the water fluxes were lower in the spacer-filled channels than the empty channels at inlet velocity 0.01 m/s. In addition, the spacer with larger center-to-center distance showed higher flux, indicating that the presence of spacer filaments reduced the water flux at the velocity 0.01 m/s. On the contrary, at inlet velocities of 0.08 and 0.15 m/s, the spacer-filled channels showed higher flux than the empty channels and the spacer with lower center-to-center distance showed higher flux.

**Figure 5:**
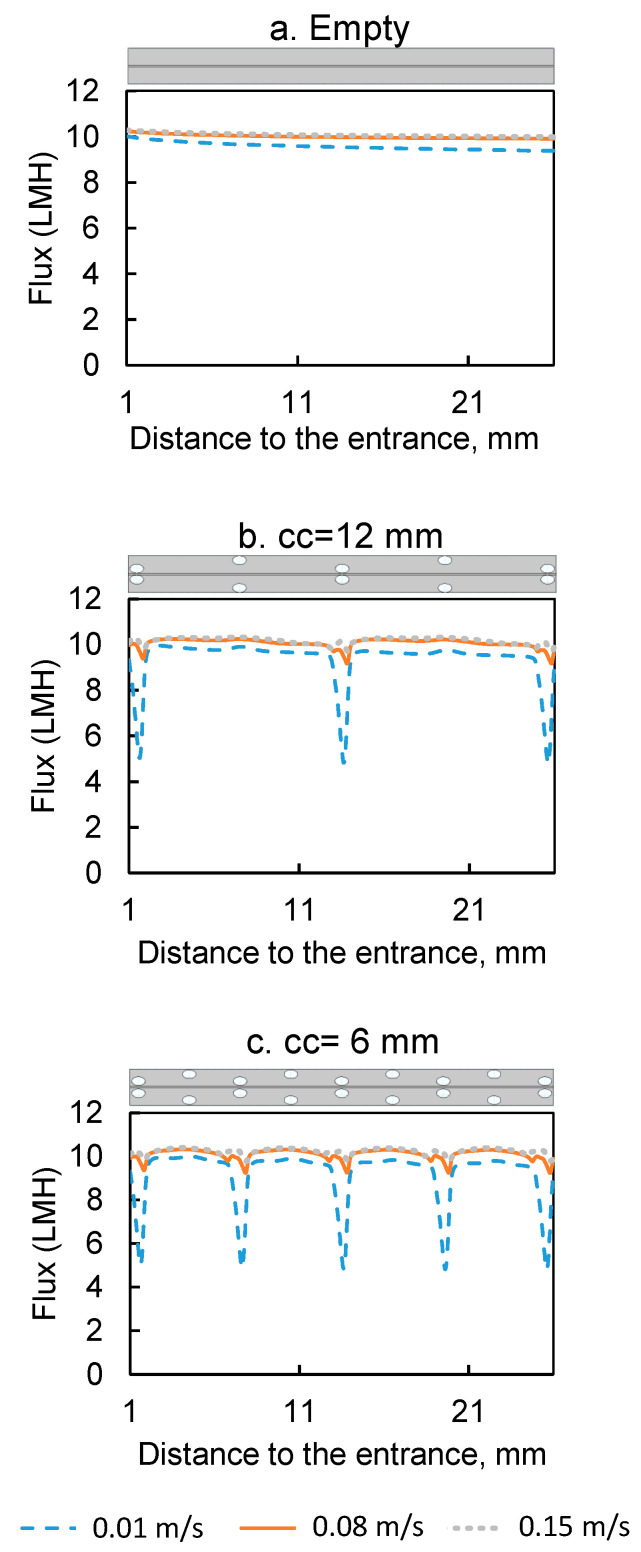
Water fluxes under different velocities along the membrane surface from the empty flow channel and the spacer-filled flow channels with center-to-center filament distances of 6 mm and 12 mm and 0.01 mm distance between the filaments and membrane. The first and last 1 mm of the channel were excluded to remove entrance and exit effects. The sketch of the part of the channel whose data are presented is placed on top of the corresponding plot to illustrate the location of the spacer inside the flow channel.

**Figure 6:**
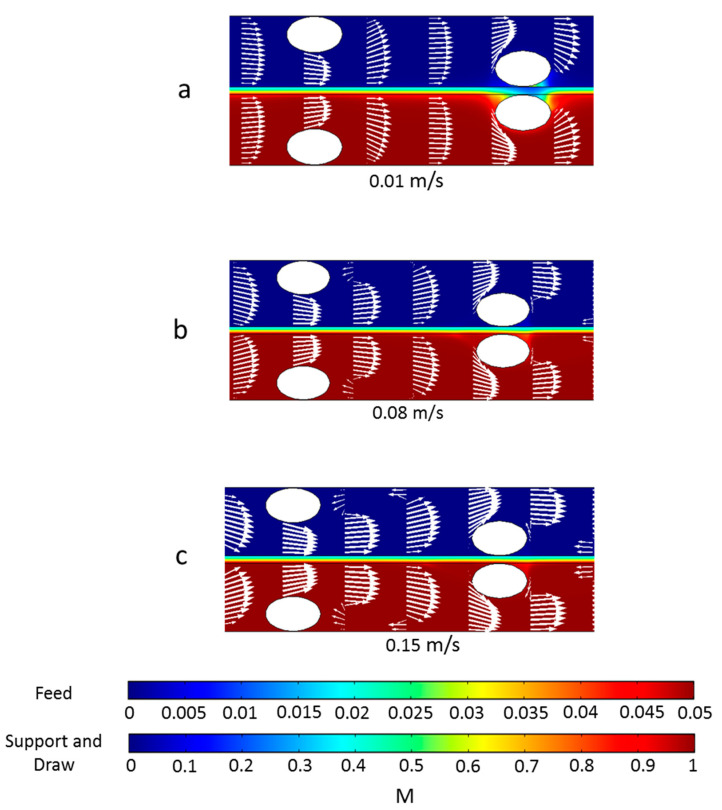
Concentration profile (indicated by the color flood) and velocity profile (indicated by the arrows) under different inlet velocities: (**a**) 0.01 m/s, (**b**) 0.08 m/s, and (**c**) 0.15 m/s. These spacers have a center-to-center filament distance of 6 mm. The size of the arrows is proportional to the logarithmic value of the velocity in order to better show vortices. Data are from the section whose distance from the entrance is approximately 14.5 to 20.5 mm.

To better understand why spacers functioned differently in flux enhancement under different inlet velocities, the local water fluxes along the membrane surface are shown in Figure 5 and the concentration and velocity profiles are plotted in Figure 6. The feed spacer filament that was close to the membrane caused flux variation in its vicinity. The local variation was much more obvious under inlet velocity 0.01 m/s (Figure 6a) than 0.08 m/s and 0.15 m/s. The flux profiles for the 0.08 m/s and 0.15 m/s inlet velocities were very similar (Figure 5). In the flow profile of the 0.08 m/s (Figure 6b) and 0.15 m/s (Figure 6c), some velocity vectors with reverse direction were observed around the spacer filaments, which indicate vortices; however, in the channel with 0.01 m/s velocity, such vortices were absent.

The concentration profiles under various velocities were different from one another and the differences primarily existed in the draw solution channel (Figure 6). First, in the channel with 0.01 m/s velocity, a dead zone was observed on the draw solution side near the feed spacer that was close to the membrane. We define a dead zone as a location where velocity is much lower than elsewhere; this is indicated by regions with very small or no white arrows in Figure 6. On the draw solution side dead zones have low concentration because there is low mass transfer of salts toward the membrane. These dead zones are also regions of high ECP in the draw-solution channel coupled with an exacerbated ICP indicated in Figure 6 by the blue color in the membrane support layer between the spacer filaments. The coupled ECP and ICP caused the flux drops near the spacers shown in Figure 5. Dead zones were much less visible in channels with 0.08 m/s and 0.15 m/s velocity, so it appears that the higher velocity was able to create vortices near the feed spacer, which minimized the dead zones near the spacer filaments.

To summarize the results from Figure 4, Figure 5 and Figure 6, when the velocity was low, spacer filaments only caused dead zones with no vortex formation, so a greater number of filaments meant greater CP (both ICP and ECP) and thus lower flux. On the contrary, when the velocity was high enough, filaments caused vortex formation that decreased the overall CP, so more filaments meant greater flux.

### 3.3. The Impact of Spacer Membrane–Filament Distance

**Figure 7:**
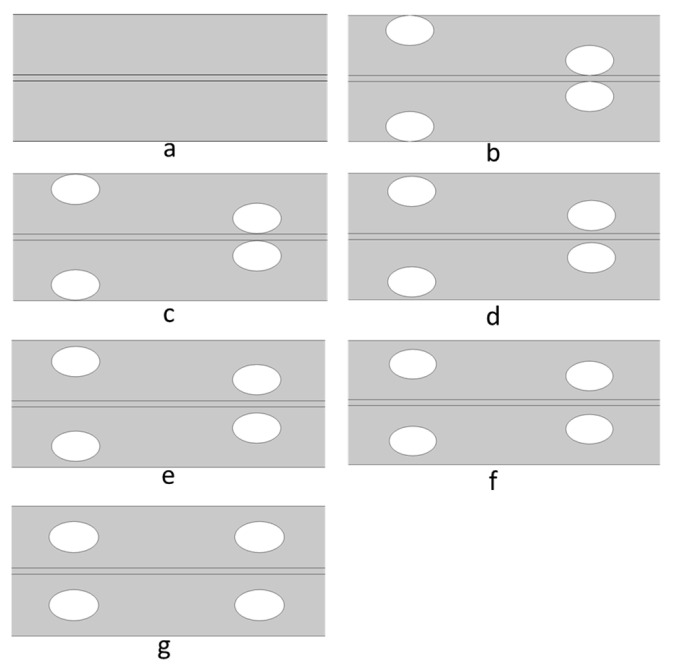
Illustration of (**a**) the empty channel, and spacer-filled membrane channels with different membrane–filament distances (**b**) 0, (**c**) 0.01, (**d**) 0.05, (**e**) 0.1, (**f**) 0.15, and (**g**) 0.25 mm. The center-to-center distance was 6 mm.

Five different membrane–filament distance scenarios were studied, where the distances were 0, 0.01, 0.05, 0.1, 0.15, and 0.25 mm. The same values were also used as wall–filament distance (for alternating filaments). As a result, the sum of the membrane–filament distance and wall–filament distance took 0%, 2%, 10%, 20%, 30%, and 50% of the channel height, respectively (Figure 7).

**Figure 8:**
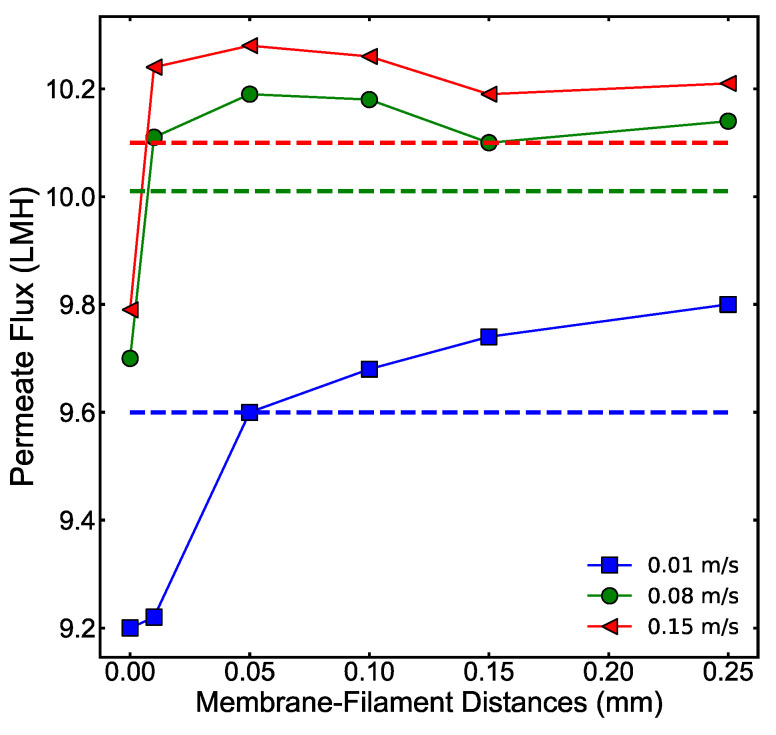
Water flux vs. membrane–filament distance for different crossflow velocities. A membrane with empty channels is used as a control and its flux is indicated with dotted lines. The spacer geometries are shown in Figure 7.

Water flux from the simulations with different membrane–filament distances is shown in Figure 8. Water fluxes for the 0 mm distance under all velocities were the lowest because of surface blockage by the spacer. At 0.01 m/s flow velocity the flux increased with the membrane–filament distance and the channels with 0.1 mm to 0.25 mm membrane–filament distance had higher flux than the empty channel. For 0.08 m/s and 0.15 m/s velocities the highest flux was observed at the 0.05 mm membrane–filament distance.

**Figure 9:**
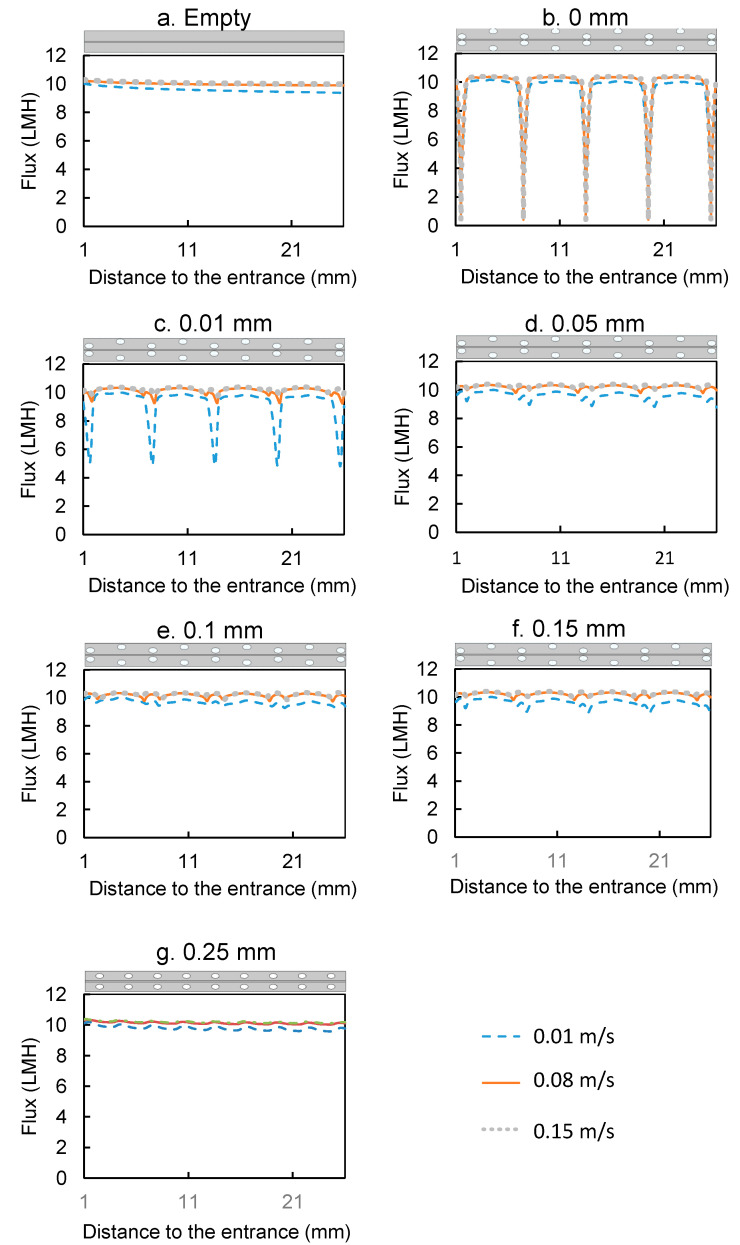
Water fluxes under different velocities along the membrane surface from the empty flow channel and the spacer-filled flow channels with various membrane–filament distances. The first and last 1 mm of the channel were excluded to remove entrance and exit effects. The sketch of the part of the channel whose data are presented is placed above the corresponding plot to illustrate the location of the spacer inside the membrane channel.

The local fluxes on the membrane surface are shown in Figure 9. The flux profiles for membrane–filament distances from 0 mm to 0.05 mm were quite distinct and suggest that the impact of the spacer became smaller as the membrane–filament distance increased; the flux profiles became flatter and tended to be more similar to one another. This is especially noted at 0.25 mm; while the average flux for 0.25 mm was a bit higher than the empty channel (as seen in Figure 8), the flux profiles in that channel were very similar to those in the empty channel, suggesting that the impact of the spacer was limited when it was furthest from the membrane.

**Figure 10:**
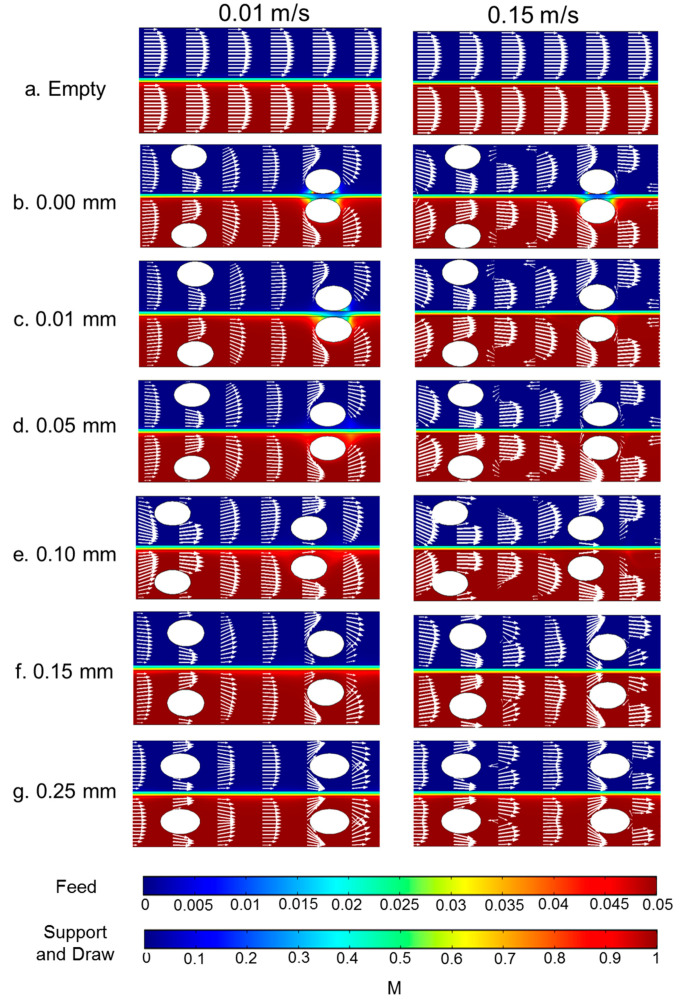
Concentration profile (indicated by the color flood) and velocity profile (indicated by the arrows) under different inlet velocities and membrane–filament distances for spacer-filled flow channels with spacers having center-to-center filaments of 6 mm. The size of the arrows is proportional to the logarithmic value of the velocity to better show the vortices. The data were from the section whose distance from the entrance is approximately 14.5 to 20.5 mm.

The velocity and concentration profiles for the 0.01 m/s and 0.15 m/s flow velocities of various membrane–filament distances are shown in Figure 10. The 0.08 m/s flow velocity is not shown but was very similar to the 0.15 m/s case. Both large membrane–filament distance and velocity reduced the dead zones around the spacer near the membrane on the draw solution side. Furthermore, higher crossflow velocity suppressed the ECP layer near the membrane. Higher crossflow velocity created vortices in spacer-filled channels; however, the vortices diminish when the membrane–filament distance increases from 0.05 mm to 0.1 mm or higher. The lack of vortices in channels with membrane–filament distances of 0.15 mm and 0.25 mm explains the reduction in water flux in those channels shown in Figure 8.

The results for crossflow hydrodynamics reported here show similar flow patterns and fluid phenomena as reported by others for spacer-filled RO channels [42,43]. Our contribution is coupling those hydrodynamics with solute transport both through the channels and within the membrane support layer to evaluate the effects of spacers on salt buildup and the resulting concentration-driven flux. It is clear from these models that spacers (or parts of spacers) that touch the membrane can increase CP (both internal and external) and decrease flux, while spacers (or parts of spacers) suspended away from the membrane can decrease CP and enhance flux.

### 3.4. 3D Simulations with Spacer Filled Flow Channels

**Figure 11:**
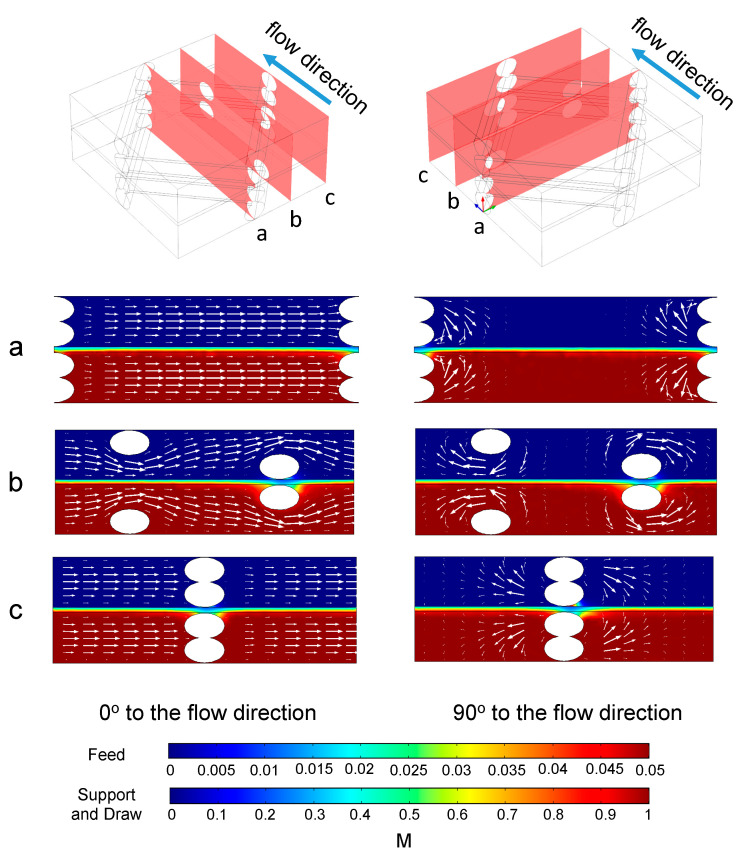
Concentration profile (indicated by the color flood) and velocity profile (indicated by the arrows) in the sections (**a**–**c**) of the 3D model corresponding to the diagrams at the top of each column. Velocity was 0.01 m/s. For each cross section, the size of the arrows is proportional to the value of the velocity.

As mentioned in Section 2.4, the fully coupled 3D simulation is limited and could only converge for 0.01 m/s flow velocity. The result of the concentration and velocity distribution from different cross sections are shown in Figure 11. In the slices perpendicular to the main flow direction flow patterns with vortices are seen near the spacer filaments, which the 2D models could not have shown.

**Figure 12:**
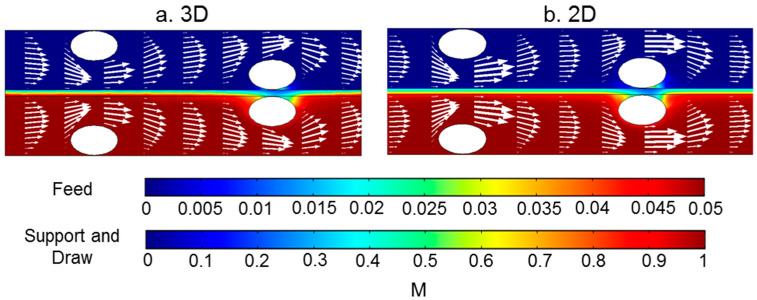
Concentration profile (indicated by the color flood) and velocity profile (indicated by the arrows) in (**a**) the middle section of the 3D model and (**b**) the section from the 2D model where the distance to the entrance was from 9.5 mm to 14.5 mm. The size of the arrows is proportional to the value of the velocity.

By comparing the results of 2D and 3D modeling in the slice similar to the 2D models, similar concentration profiles were found (Figure 12). The velocity profiles from the two models were similar in the region between spacer filaments. However, there was a notable difference in velocity magnitudes in the areas above and below the spacer filaments. In the 2D model the velocities above and below the spacer filaments were higher than those calculated in the 3D model. This is because in the 3D model the flow was able to travel around the filaments in multiple directions, but in 2D the flow was forced through the narrowed section of the channel.

**Figure 13:**
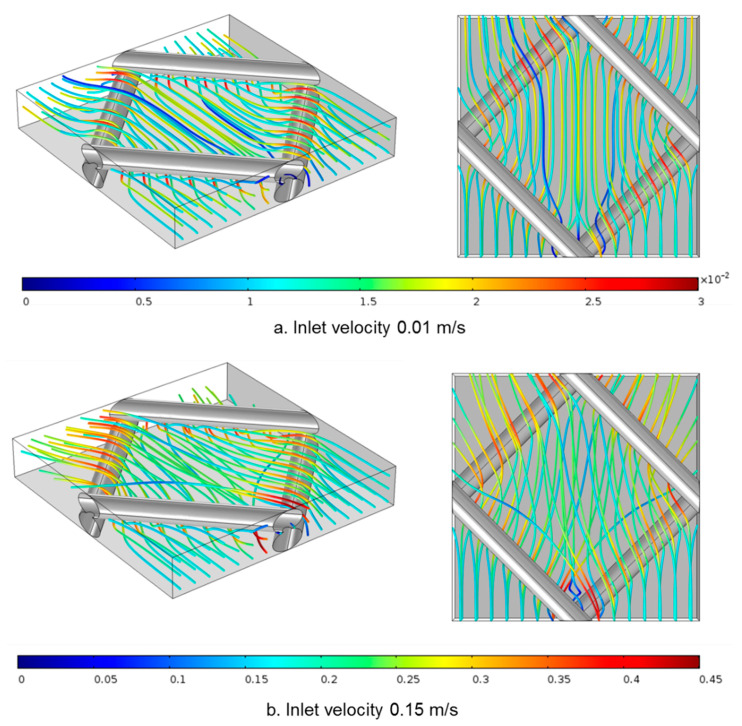
Velocity profiles inside 3D spacer-filled channels. The flow path is indicated by the streamline and the local velocity value is indicated by the color. Two inlet flow rates were tested: (**a**) 6 × 10^–7^ m^3^/s (corresponding to inlet velocity of 0.01 m/s) and (**b**) 9 × 10^−6^ m^3^/s (corresponding to inlet velocity of 0.15 m/s). For each flow rate, an orthogonal view (left) and top view (right) are provided.

To reveal more details of the flow in the 3D model, the flow paths inside the spacer-filled membrane channel under different flow velocities are shown in Figure 13. This model focused on fluid flow without considering the mass transfer of the salt and water flux, as others have done [30]. It has been reported that crossflow hydrodynamics in spacer-filled RO channels are not greatly affected by the flux [44,45]. Because the water flux was only 0.2% of the inlet flow rate, neglecting the water flux did not have a significant impact on the flow pattern inside the channels.

The streamlines show how water flows around the spacer: the flow paths travel over and under the filaments, but also deviate left and right to find paths of least resistance. Increasing inlet velocity enhanced the 3D flow patterns as we observed higher degrees of flow path cross-over with 0.15 m/s than 0.01 m/s flow velocities. These results show that the 3D model was more suitable to capture the 3D pattern of the flow inside spacer-filled flow channels, especially in high crossflow velocities.

## 4. Conclusions

Fully coupled multiphysics CFD models predicted experimental water flux in empty flow channels of FO and PRO. This is a step forward in membrane modeling because most previous efforts used hydraulic pressure difference or assigned water flux during simulations based on experimental data. Here water flux was truly predicted—it depended only on solute concentrations that induced an osmotic pressure driving force.

Both 2D and 3D CFD models showed that spacers created vortices inside the flow channel. Increasing flow velocity augmented the vortices and improved flux; however, there were diminishing returns as inlet velocity increased. The study also showed that increasing the membrane–filament distance reduced the dead zones in the vicinity of the spacer. This positive effect was mitigated by a reduction in vortices and decreased water flux as membrane–filament distance increased. In these simulations, a 0.05 mm membrane–filament distance was optimal.

The information garnered from 2D models deviated from reality to some degree. 2D models did shed light on the general trends in behavior (such as general changes in flux as the membrane–filament distance changed), but 3D models were able to show more detailed flow and concentration profiles because they could explore all possible flow angles. These fully coupled 3D models were resource intensive, so further work at improving modeling capabilities is warranted, including modifying the coupling methods between fluid flow and solute transport such that solutions can be found even for 3D simulations of complex spacer geometries.

## Figures and Tables

**Table 1:** Boundary conditions for ODMP simulations. The location of each boundary is depicted in Figure 1.

Boundary Number	Fluid Flow			Solute Mass Transport		
	Feed Channel	Porous Support	Draw Channel	Feed Channel	Porous Support	Draw Channel
1	Inlet (*u_f_*)			Inlet (*c_f_*)		
2	Outlet Atm. Pressure			Outlet Atm. Pressure		
3	Outlet (*J_w_*) No-slip parallel to membrane	Inlet (*J_w_*)		Inlet (*J_s_*)	Outlet (*J_s_*)	
4		Outlet (*J_w_*)	Inlet (*J_w_*) No-slip parallel to membrane		Inlet (*J_s_*)	Outlet (*J_s_*)
5			Inlet ($u_{d}$)			Inlet (*c_d_*)
6			Outlet Atm. Pressure			Outlet Atm. Pressure
7	Impermeable No-slip			Impermeable No-slip		
8			Impermeable No-slip			Impermeable No-slip

**Table 2:** Standard deviations of water flux (L/m^2^/hr) in the literature data.

	FO #1		FO #2		PRO #1		PRO #2	
	TFC	Asymmetric	TFC	Asymmetric	TFC	Asymmetric	TFC	Asymmetric
Lab A	3.13	1.32	2.66	1.96	3.67	1.50	3.21	1.34
Lab B	0.14	0.97	0.43	0.16	1.83	0.10	1.16	0.04
Lab C	1.35	0.35	3.24	1.15	4.37	0.76	0.68	0.07
Average	1.54	0.88	2.11	1.09	3.29	0.79	1.69	0.48

## List of Symbols

$A_{F}\;$	Water permeability of the membrane at 293 K, m/(s·Pa)
B	Salt permeability of the membrane, 1/s
*c*	Salt concentration, M
$c_{d}$	Inflow salt concentration at the draw solution side, M
$c_{f}$	Inflow salt concentration at the feed solution side, M
D	Diffusion coefficient in the membrane channel, m^2^/s
$D_{e}$	Effective diffusion coefficient in the membrane support layer, m^2^/s
$J_{w}\;$	Flux of water, m/s
$J_{s}\;$	Flux of salt through the membrane, M·m/s
L	Length of the channel, mm
*P*	Pressure, Pa
R	Salt rejection ratio
u	velocity, m/s
$u_{d}$	Inflow velocity at the draw solution side, m/s
$u_{f}$	Inflow velocity at the feed side, m/s

## Greek Letters

ε	Porosity of the membrane support layer
τ	Tortuosity of the membrane support layer
κ	Hydraulic conductivity, m/s
ρ	Density, kg/m^3^
μ	Dynamics viscosity, Pa·s
$\beta_{F}$	Forchheimer coefficient
$\pi_{d}$	Osmotic pressure, draw solution side nearest the membrane, Pa
$\pi_{f}\;$	Osmotic pressure, feed solution side nearest the membrane, Pa
γ	Shear rate, 1/s
